# An Integrated Strategy for Nutraceuticals from *Haematoccus pluvialis*: From Cultivation to Extraction

**DOI:** 10.3390/antiox9090825

**Published:** 2020-09-03

**Authors:** Sanjeet Mehariya, Neeta Sharma, Angela Iovine, Patrizia Casella, Tiziana Marino, Vincenzo Larocca, Antonio Molino, Dino Musmarra

**Affiliations:** 1 Department of Engineering, University of Campania “Luigi Vanvitelli”, Real Casa dell’Annunziata, Via Roma 29, 81031 Aversa (CE), Italy; sanjeet.mehariya@unicampania.it (S.M.); angela.iovine@unicampania.it (A.I.); tiziana.marino@unicampania.it (T.M.); 2 ENEA, Italian National Agency for New Technologies, Energy and Sustainable Economic Development, Department of Sustainability-CR Portici, P. Enrico Fermi, 1, 80055 Portici (NA), Italy; patrizia.casella@enea.it (P.C.); antonio.molino@enea.it (A.M.); 3 ENEA, Italian National Agency for New Technologies, Energy and Sustainable Economic Development, Department of Sustainability-CR Trisaia, SS Jonica 106, km 419 + 500, 75026 Rotondella (MT), Italy; neeta.sharma@enea.it (N.S.); vincenzo.larocca@enea.it (V.L.)

**Keywords:** microalgae, astaxanthin, lutein, fatty acids, antioxidant, extraction, algal extract

## Abstract

The aim of this study was to develop an effective integrated cultivation system for *Haematococcus pluvialis* as a source of bioactive compounds such as astaxanthin, lutein, proteins, and fatty acids (FAs). The Chlorophyta *H. pluvialis* was cultivated in a vertical bubble column photobioreactor (VBC-PBR) under batch mode, allowing switching from green to red phase for astaxanthin induction. The combined effect of light intensity and nutrients on bioactive compound formation was investigated. Results showed that growth under lower nutrients availability and light intensity led to a higher concentration of biomass. Growth under high light intensity with an appropriate concentration of nitrate, sulfate, phosphate and magnesium led to ~85% and ~58% higher production of total carotenoids and fatty acids, respectively. Under high stress conditions, ~90% nitrate and phosphate consumption were observed.

## 1. Introduction

Microalgae are eukaryotic photosynthetic microorganisms that can grow in brackish water, fresh water, and sea water. During photosynthesis, microalgae use carbon dioxide (CO_2_) as a carbon source and solar energy is converted into chemical energy to produce new biomass. Microalgae can be considered as potential bio-factories able to capture and use high amounts of CO_2_ (10–50 times more than terrestrial plants) and to produce high-value compounds [1].

*H. pluvialis* is a promising source of bioactive compounds like carotenoids, proteins, and fatty acids (FAs), in particular astaxanthin, a powerful antioxidant [2]. *H. pluvialis* is an unicellular freshwater biflagellate green microalga that belongs to the class *Chlorophyceae*, order *Volvocales*, and family *Haematococcaseae* [3]. The life-cycle of *H. pluvialis* consists of two phases: the first is known as the “green vegetative phase”, while the second is referred to as “red non-motile encysted phase” and occurs under stress conditions [4,5]. During the life-cycle of *H. pluvialis* cells, ultra-structural shifts occur in the switch from the green to the red phase. The chemical composition of the cellular content also changes. During the “green phase” up to 1% of lutein and around 20–25% of fatty acids on dry biomass weight (DBW) can accumulate, while in the “red phase” around 1–5% of astaxanthin and 32–37% of lipids on DBW [3,6] can accumulate. Under high stress conditions, 5% of astaxanthin on DBW [7] can be reached and the red phase of astaxanthin can lead to up to 7.72% DW (dry weight) under the supply of 5% of CO_2_ [5,8]. High value-added compounds like astaxanthin, lutein and beta-carotene, and fatty acids offer several health benefits. Among the carotenoids, astaxanthin and lutein are well recognized as natural antioxidants. Lutein is a pigment present in the macula of the eye. Its concentration in the ocular tissue protects eyes health and can reduce the risk of age-related macular degeneration [9]. Astaxanthin is 50 times more powerful as an antioxidant than vitamin E and C [10]. Also, astaxanthin is an anti-inflammatory with therapeutic effects against several human diseases like photooxidation, inflammation, cancer, *Helicobacter pylori* infection, and aging and age-related diseases [11]. Furthermore, astaxanthin is recognized as protective against UV-light, and beneficial for the liver, heart, and skin [12]. In 1999, the United States Food and Drug Administration approved astaxanthin and lutein as feed additives for uses in the aquaculture industry and as dietary supplements in the nutraceutical industry [13]. Dietary guidance was established for lutein with an acceptable daily intake (ADI) of 0–2 mg/kg body weight (bw), encouraging the consumption of lutein-containing foods and raising public awareness about its potential health benefits [14,15]. Acceptable daily intake (ADI) of astaxanthin of 0.2 mg/kg bw per day was established. According to the European Commission Implementing Regulation (EU) 2017/2470, astaxanthin-rich oleoresin derived from *Haematococcus pluvialis* can be assimilated at rates of up to 40–80 mg/day in food supplements [16]. Saturated fatty acids, as stearic acid, decrease cholesterol level, and monounsaturated and polyunsaturated fatty acids decrease the cardiovascular risk [17]. Essential omega-3, as α-linolenic and linoleic acids need to be assimilated as precursors of omega-3 DHA, EPA, and omega-6 arachidonic acid since a balanced ratio between ω-3/ω-6 is fundamental to reduce inflammatory and cardiovascular diseases [18]. Therefore, microalgae could be a viable source of these compounds to be used in food, nutraceutical, and pharmaceutical industries.

Microalgae cultivation is affected by several factors, e.g., temperature, pH, light intensity, photoperiod, reactor design, and hydrodynamic factors such as flow rate, mixing, and mass transfer of CO_2_ in growth medium; nutrients concentration (nitrogen (N) and phosphorus (P)) is also essential for the cell development and its metabolic activity. The combination of these factors could significantly influence the biomass production, the intracellular composition, and the chlorophyll content [19,20]. Chlorophyll a and b are priority pigments for monitoring biomass growth in *Chlorophyta* microalgae e.g., *H. pluvialis* since both chlorophylls adsorb different wavelengths in UV-visible spectrum [21]. Although open pond reactors are simple and cost-effective systems for scaling up the microalgae cultivation, several drawbacks should be resolved such as water loss from medium due to evaporation, low growth rate due to improper light and CO_2_ mass-transfer, and contamination. Therefore, closed photobioreactors (PBRs) such as bubble column photobioreactors (PBRs), which assure a homogeneous CO_2_ mass-transfer and light intensities distribution, could be the most suitable choice for the cultivation of microalgae [22], and also for reusing/recycling the culture medium, thus allowing us to reduce water consumption [23].

*Haematococcus pluvialis* growth was investigated in two steps to enhance biomass and astaxanthin production in the green and red phase, respectively [24,25,26]. Fábregas et al. [27] observed the highest cell density in the green phase after 6 days, while the highest astaxanthin accumulation was recorded in the red phase when cultures were exposed to high light intensity after 15 days. Aflalo et al. [28] compared one- and two-stage approaches highlighting the highest astaxanthin yields, productivity, and process efficiency in two-stage cultivation. Furthermore, some authors demonstrated the production of astaxanthin from *Haematococcus pluvialis* in a one-stage continuous mode in regulating nitrate uptake [29]. Several stress factors were investigated to increase astaxanthin production in *H. pluvialis* red phase [30]. Nutrient depletion and high light intensity are the most effective stress factors to enhance astaxanthin production in the red phase after green phase cultivation at low light intensity and balanced supply of nutrients [31,32]. Nitrogen depletion coupled to high light intensity was found to be more efficient than phosphorus deficiency since the lack of nitrogen affects chlorophyll deterioration [33]. *Haematococcus pluvialis* cultivation was also performed by supplying carbon dioxide in the green phase to improve biomass production [34,35]. The gradual increase scalability of the PBRs system and the hydrodynamic performance of carbon dioxide from flue gas was also investigated [36,37]. Another factor determining production of *H. pluvialis* biomass and astaxanthin is light intensity [38]. Blue and red LED lights were investigated as alternative light sources for enhancing astaxanthin production in the red phase [39,40]. Some studies focused on the green phase cultivation by using batch-stage, fed-batch stage, and inoculum replacement [41] or using different inoculum percentages to promote biomass production [42].

Research aims at overcoming some disadvantages of *H. pluvialis* and astaxanthin production, such as increasing growth in the green stage, simplifying the two-stage cultivation, and implementing the production and extraction of astaxanthin and other high value compounds [43]. Furthermore, integrated strategies are being investigated combining effective two-stage cultivation [44], biomass pre-treatment [45], and extraction technologies such as green solvent and supercritical fluid extraction using carbon dioxide [46,47].

In this work, an integrated approach was investigated using *H. pluvialis* cultivated in a bubble column PBR for the production of astaxanthin, lutein, and FAs. The effect of re-using media was investigated during green phase and different light intensities were explored during the red stage. Subsequently, an optimal extraction method was investigated using green chemistry for the extraction of astaxanthin, lutein, and FAs.

## 2. Materials and Methods

### 2.1. Microalgae and Growth Medium

The microalgae *H. pluvialis* seed culture was collected from a commercial producer of *H. pluvialis* biomass (AlgaRes Srl, Rome, Italy), and used for cultivation under laboratory conditions. Microalgae cells were cultured in BG-11 (Blue-Green) medium [36] consisting of EDTA disodium (2.7 × 10^−9^ mM), NaNO_3_ (1.8 × 10^−5^ mM), K_2_HPO_4_ (2.3 × 10^−7^ mM), MgSO_4_·7H_2_O (6.2 × 10^−7^ mM), CaCl_2_·2H_2_O (2.4 × 10^−7^ mM), C_6_H_8_O_7_ (3.12 × 10^−8^ mM), C_6_H_8_FeNO_7_ (2.29 × 10^−8^ mM), and Na_2_CO_3_ (1.89 × 10^−7^ mM). A micronutrient solution (10 mL) containing H_3_BO_3_ (4.63 × 10^−5^ mM), MnCl_2_·4H_2_O (9.15 × 10^−6^ mM), ZnSO_4_·7H_2_O (7.72 × 10^−7^ mM), Na_2_MoO_4_·2H_2_O (1.89 × 10^−6^ mM), CuSO_4_·5H_2_O (3.16 × 10^−7^ mM), and Co(NO_3_)_2_·6H_2_O (1.70 × 10^−7^ mM) was also added to 990 mL of BG-11 medium. The *H. pluvialis* inoculum was sub-cultured in the BG-11 medium at 28 °C, 250 µmol photons/m^2^/s using a white fluorescent tube light bulb white, 3000K (Philips, Amsterdam, The Netherlands).

### 2.2. Vertical Bubble Column Photobioreactor Conditions

**Figure 1:**
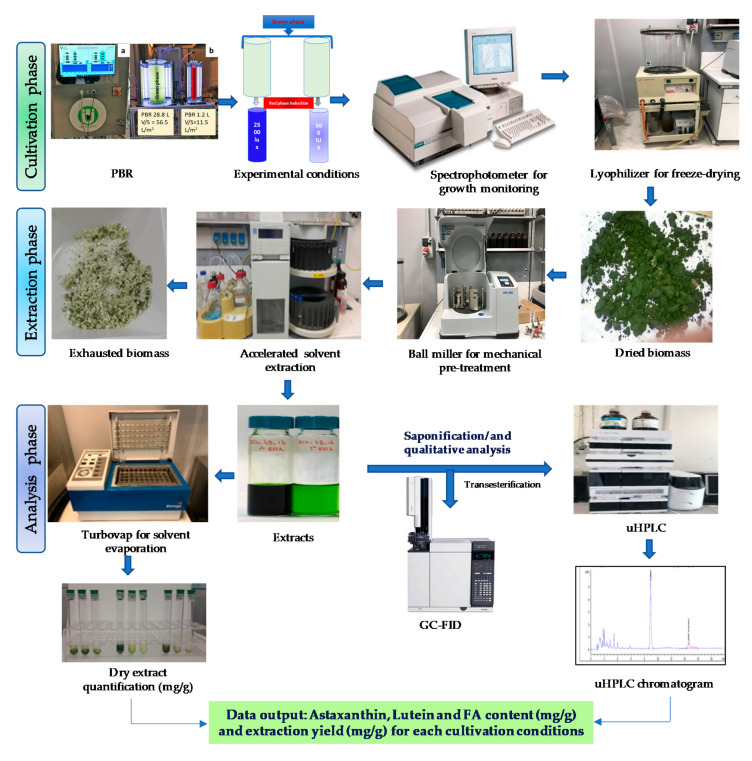
Experimental design.

*H. pluvialis* green and red phases were cultivated in two vertical bubble column photobioreactors (VBC-PBRs), in plexiglass with a volume/surface ratio (V/S) of 56.5 and 11.5 L/m^2^, respectively, and equipped with control and monitoring systems (Figure 1) (BetaSystem, Naples, Italy). VBC-PBR with V/S of 56.5 L/m^2^ had a working volume of 28.5 L (height: 680 mm; external diameter: 250 mm; thickness: 10 mm). PBR was equipped with 6 filleted holes (1/2”) at the bottom, where 6 sintered steel gas spargers were installed. The working volume was 1.25 L for VBC-PBR with V/S of 11.5 L/m^2^ measuring height: 680 mm; external diameter: 60 mm; thickness: 10 mm. The bottom of PBR was equipped with 3 filleted holes (1/8”) and 3 sintered steel spargers were installed. The top of both PBRs had 3 holes for temperature/pH probe, and temperature control systems included an AISI 316L coaxial pipe. The coaxial pipes had a diameter of 60.3 mm and 12 mm for PBR with V/S of 56.5 L/m^2^ and PBR with V/S of 11.5 L/m^2^, respectively. Cooling water would flow inside the coaxial pipe in order to control the temperature of the photobioreactors in the range of 15–35 °C. The aeration flow rate of both PBRs could be maintained at 0–300 mL/min with flow control accuracy of 0.5% using the Bronkhorst controllers (Berkelland, The Netherlands). PBRs were also equipped with a lighting system, consisting of semi-cylinder, located at a distance of 100 mm from the PBRs with blue, white, and red lights from a selective LED system (only blue/only white/only red or a mix of them), with a light intensity of 500–5000 lux (3000 K) on the surface of PBR. The diameter of the lighting system for PBR with V/S of 56.5 L/m^2^ was equal to 350 mm, while the diameter of the lighting system for PBR with V/S of 11.5 L/m^2^ was 160 mm and lighting systems are controlled and regulated by SCADA (Supervisory Control and Data Acquisition). Temperature and pH were monitored in real time by SCADA in a user interface consisting of a custom software and PC with touchscreen [48,49].

### 2.3. Cell Growth Measurements

*H. pluvialis* cell growth was monitored by determining the absorbance of samples at 420 (Chlorophyll-a), 480 (Chlorophyll-b), 690 (Chlorophyll-a), and 620 nm (Chlorophyll-b) using a UV/Visible spectrophotometer (Multiskan, Thermo Fisher Scientific, Waltham, MA, USA). The morphology changes were randomly monitored under an optical microscope (Nikon Instruments Inc, Melville, NY, USA) at 400× magnification.

The biomass dry weight (*BDW*) was calculated using the absorbance values at different biomass concentrations evaluated during the green phase, obtaining a calibration line between absorbance and concentration as showed below:

(1) $$BDW=\left(0.0867*A\right)-0.1868$$

where: *BDW* is the concentration of biomass dry weight (g/L), *A* is total absorbance obtained summing the absorbance values at 420 nm and 690 nm of chlorophyll-a and at 480 nm and 620 nm of chlorophyll-b.

For final dry weight determination, cell cultures were dewatered by vacuum filtration system using vacuum filters with a pore size of 0.45 μm (Sigma-Aldrich, St. Louis, MO, USA) and the pellets were lyophilized for 24 h by using Edwards Lyophilizer (©Edwards, Hillerød, Denmark).

### 2.4. Growth Conditions and Inoculum Reuse

*Haematococcus pluvialis* was cultivated during the green phase in VBC-PBR with the volume/surface ratio (V/S) of 56.5 L/m^2^ (Figure 1). BG-11 medium was used for the cultivation following the aforementioned concentration (Section 2.1). Green phase cultivation was performed at 28 °C, pH 7.5–8.5 °C, using the white light LED (4000 lux intensity, 100 µmol/photons/m^2^/s) with an air flow rate of 300 mL/min. pH was measured. At the beginning of the stationary phase a second green phase was started by re-using around 30% of *H. pluvialis* culture. About 8.5 L of *H. pluvialis* culture was mixed with 19.5 L of BG-11 medium (~1:3 ratio) to perform the second green phase growth. At the beginning of both green phase cultivations, *Haematococcus pluvialis* inoculum had an optical density (OD) and a dry weight equal to OD~2 and around ~1.5 g/L, respectively.

Red phase was performed in VBC-PBR with the volume/surface ratio (V/S) of 11.5 L/m^2^ at 28 °C, pH 7.5–8.5 °C, with an air flow rate of 50 mL/min. Red phase cultivation was carried out testing two different light intensities at 500 lux (55 µmol/photons/m^2^/s), and 2500 lux (280 µmol/photons/m^2^/s).

### 2.5. Accelerated Solvent Extraction

Bio-compounds such as carotenoids and fatty acids were extracted from *H. pluvialis* lyophilized cells. The biomass was mechanically pre-treated through a planetary ball mill PM 200 (Retsch GmbH, Haan, Germany) and extraction was performed by using Accelerated solvent extractor, ASE 200 Dionex© (Salt Lake City, UT, USA). The pretreatment and the extraction were carried out at optimum conditions as described in previous work [50]. Extraction was performed using ethanol at 67 °C and 10 MPa. Four consecutive extraction cycles were performed for a total extraction time of 80 min to complete discoloration of biomass. At the end of each extraction run (20 min), the system flushes 6.6 mL of fresh solvent, and nitrogen (Purity ≥ 99.999%) was purged for 1 min. Around 20 mL of extract was obtained and were collected into 40 mL amber glass vials. The extracts of *H. pluvialis* red phase biomass were equally transferred into three different vials for gravimetric analysis after the drying process by using a Zymark TurboVap evaporator (Zymark, Hopkinton, MA, USA).

### 2.6. Analytical Methods

Anions (NO_3_^−^, NO_2_^−^, Cl^−^, PO_4_^3−^) and cations (Mg^2+^, SO_4_^2−^, Na^+^, Ca^2+^, K^+^) concentration for each growth phase (1st and 2nd green and red phases) were analyzed using an ion Chromatograph (Dionex™ ICS-1100, Thermo Scientific, Waltham, MA, USA). The Dionex ICS-1100 was an integrated ion chromatography system equipped with a pump, injection valve, and conductivity detector. Anions were detected using a column IonPac AS14 (Dionex™, Sunnyvale, CA, USA) (250 × 4 mm) and eluent 3.5 mM sodium carbonate/1 mM sodium bicarbonate was used at a flow rate equal to 1.2 mL/min. Cations were analyzed using column IonPac SCS 1 (Dionex™) (250 × 4 mm), and eluent 3 mM oxalic acid was used at a flow rate of 1 mL/min. A mixture of combined anions and cations was used as standard (P/N 057590, P/N 046070, Thermo Scientific™ Dionex™).

For fatty acid analysis, a known amount (5 mL) of extracts were trans-esterified in two steps using methanolic sodium hydroxide solution (NaOH 0.5 M) as the alkali catalyst and boron trifluoride (BF_3_) methanol solution (14%) as the acid catalyst according to the standard method [51]. After transesterification, isooctane was added to separate FAMEs, and the upper layer (1–2 mL) was transferred to a GC glass vial. The chromatographic analysis was carried out using a 7820A GC-FID equipped with an HP-88 100 mt × 0.25 mm × 0.2 µm column. The chromatographic injector temperature was maintained at 250 °C and column was heated at 150 °C for 5 min. For oven temperature programming, temperature increased to 180 °C ramping at 1.6 °C/min, then at 1.4 °C/min to 190 °C, and finally holding the temperature at 190 °C for 10 min, as described in standard methods [52]. Nitrogen (purity 99.9999%) was used as carrier gas with a linear velocity of 30 cm/s and split ratio 1:100. The FAs characterization was carried out after each cycle extraction and heneicosanoic acid (C:21) was used as internal standard for the quantification of fatty acid methyl esters. A mixture of 37 fatty acid methyl esters (C4–C24) (Supelco FAME 37, CRM47885) was purchased from SIGMA-Aldrich, (St Louis, MO, USA) for the quali-quantitative analysis.

For astaxanthin, lutein, and beta-carotene analysis, 5 mL of extract were saponified by adding 1 mL of NaOH solution in methanol (0.05 M) for 7 h in inert atmosphere [53]. Saponification was carried out in order to remove lipids and chlorophylls, avoiding the overlap of the spectra with the carotenoids. Ammonium chloride (NH_4_Cl) solution in methanol (0.05 M) (3 mL) was added to stop saponification. Astaxanthin, lutein, and carotene were measured using u-HPLC Agilent 1290 Infinity II (Santa Clara, CA, USA) with Zorbax reverse phase C18 column with methanol-water (95:5, *v*/*v*) as a mobile phase solvent. Before the analysis, the sample was dissolved in a mixture of methanol/chloroform (90:10 containing 0.1% BHT as antioxidant agent). The flow rate and column temperature were kept constant at 0.4 mL/min and 28 °C, respectively as described in our earlier publication [54,55,56]. Statistical analysis was done by using Past (free-software). An Anova Kruskal-Wallis test was performed on several samples, and three replicates were considered. Significant results had a *p*-value < 0.05.

## 3. Results

**Figure 2:**
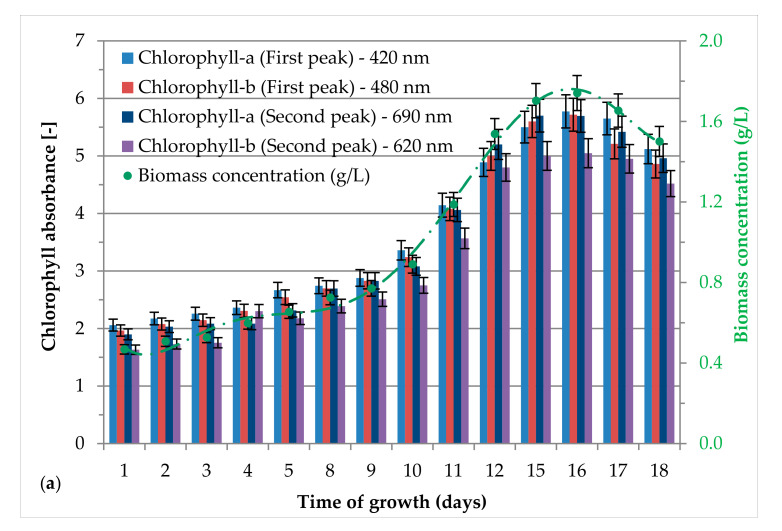

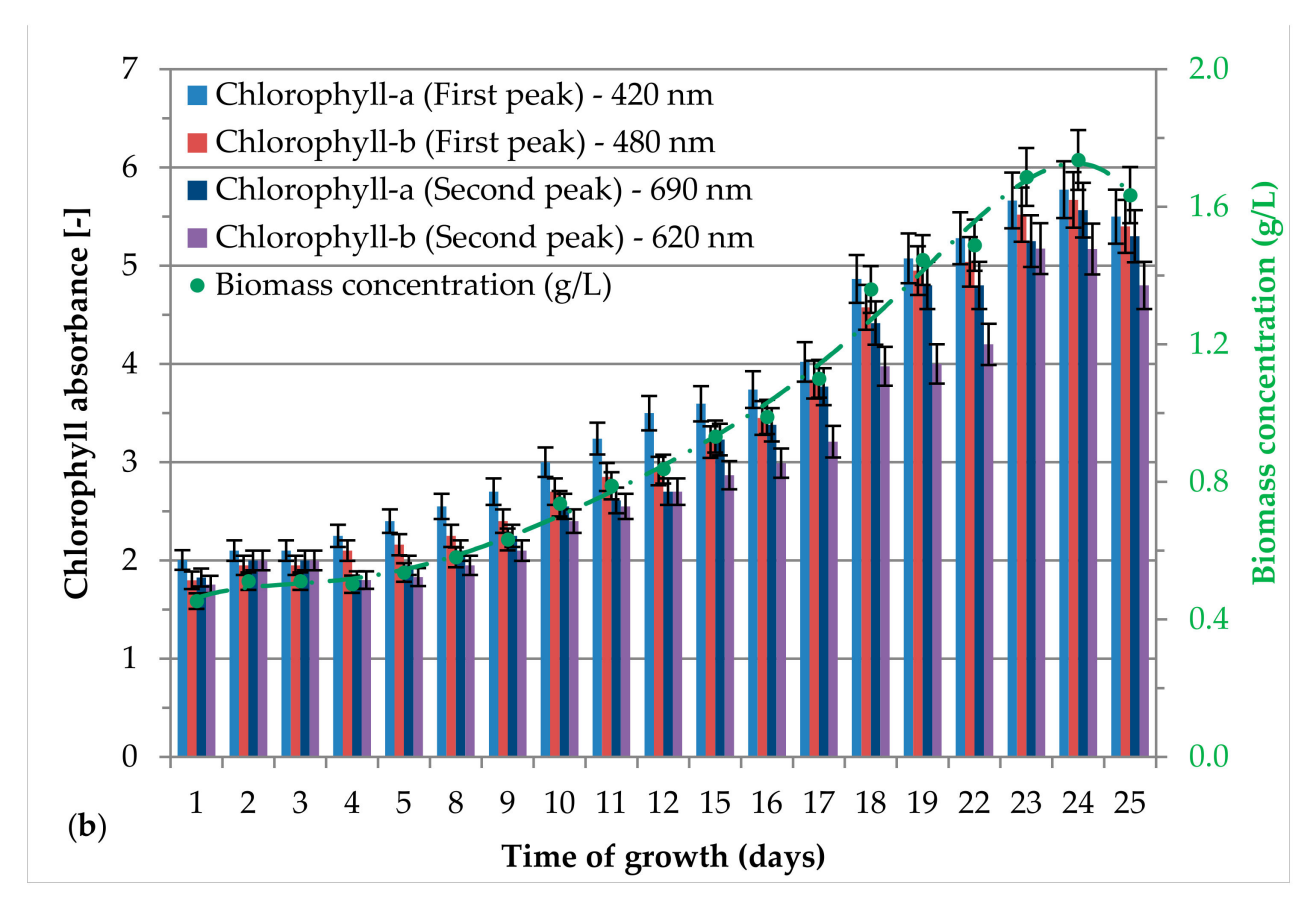
Chlorophyll absorbance and biomass concentration during the growth phases for *H. pluvialis* in green phase: (**a**) First growth with fresh inoculum, (**b**) Second growth with reused inoculum.

The process for the cultivation of *H. pluvialis* was performed during the green phase in a VBC-PBR with a V/S of 56.5 L/m^2^. *H. pluvialis* green phase have achieved the highest biomass concentration on day 16 which is equal to around 1.7 g/L. At the same time, absorbance of both chlorophyll-a and chlorophyll-b reached their highest values, confirming the highest concentration (Figure 2a). After inoculum reutilization for a second green phase, the same concentration of biomass was achieved on day 24. The second green phase resulted prolonged from 0 to 25 days respect to the first one. The first green phase was carried out using the initial inoculum and fresh prepared BG-11 growth medium. For the second green growth, a part of microalgae culture deriving from the first cycle was mixed with fresh medium (~1:3 ratio, around 30%).

### 3.1. Effect of Nutrients during the Growth of H. pluvialis Green Phase

**Figure 3:**
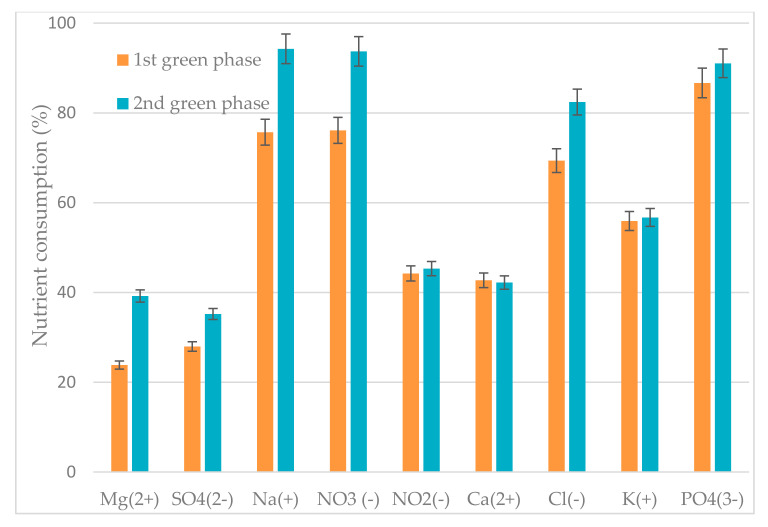
Nutrients consumption efficiency during the growth of *H. pluvialis* in the green phase.

To better understanding the performance of the green phase after inoculum reuse, nutrient concentrations (Table 1) and nutrient consumption (Figure 3) were monitored in the liquid phase. A comparison between the initial concentration of nutrients in the first and second green phase highlighted that nutrients are more abundant at the beginning of the first one. Although nutrients are lower before the second one, only Na^+^, NO_3_^−^, Ca^2+^, and Cl^−^, show a significant difference between the 1st and 2nd green phases (*p* < 0.05).

Figure 3 shows that the consumption percentage of nutrients was strictly dependent on the starting concentration. Nutrient consumption was higher in the 2nd phase than in the 1st one where high nutrients availability was recorded. Sodium, nitrate, and phosphate nutrients were the most consumed nutrients during the 2nd phase, while orthophosphate was less consumed (Figure 3).

### 3.2. Effect of Nutrients and Light Intensity during the Switch from Green to Red Phase

**Figure 4:**
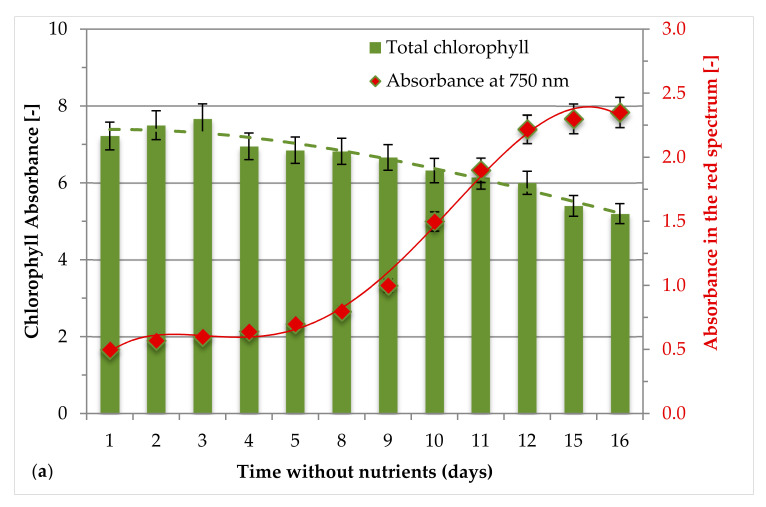

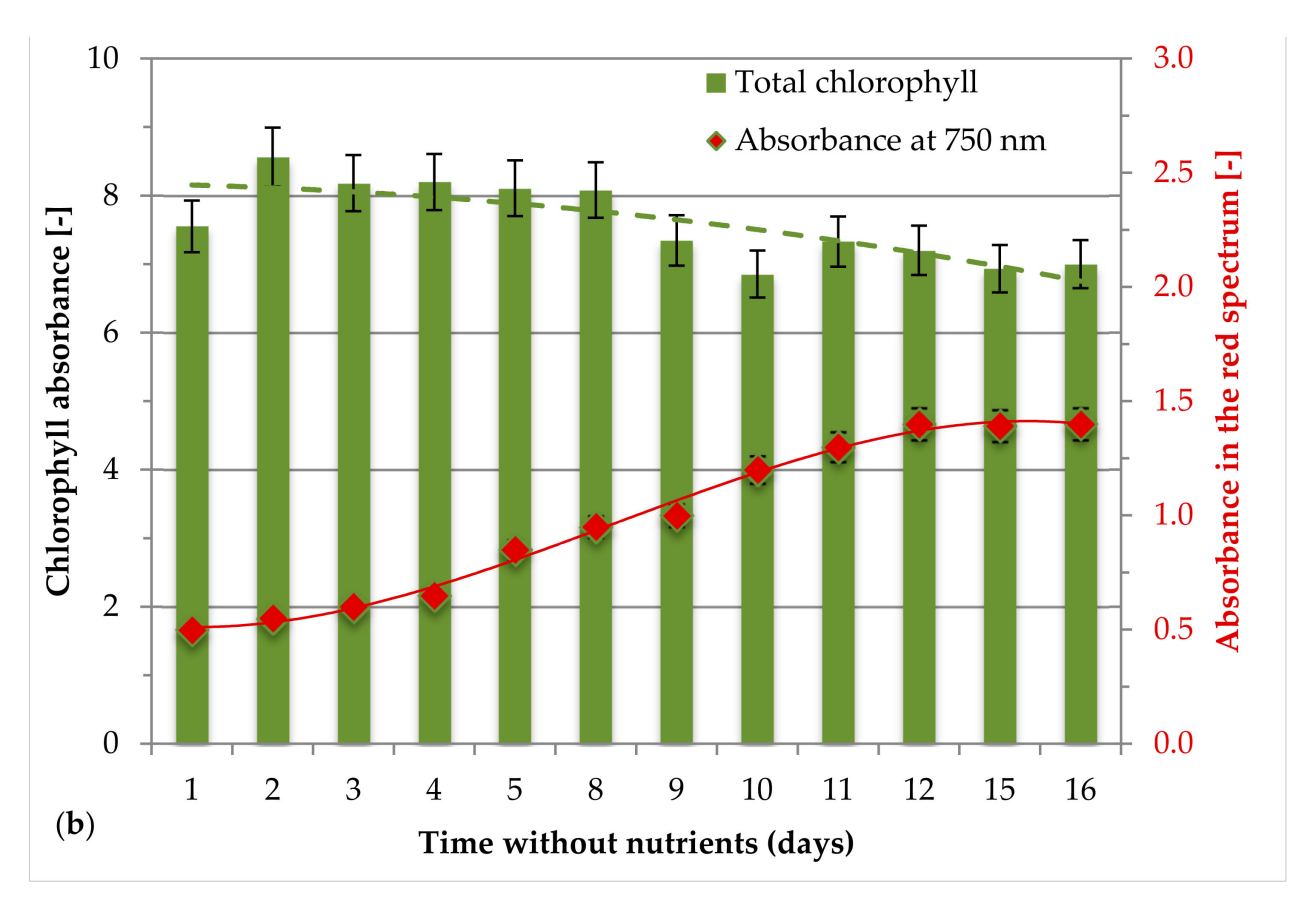
Chlorophyll absorbance during the growth phases for *H. pluvialis* red phase: (**a**) switch from green to red phase at 2500 lux (280 µmol/s/m^2^) blue light, (**b**) switch from green to red phase at 500 lux (55 µmol/s/m^2^) of blue light.

*H. pluvialis* red phase was carried out in VBC-PBR with a V/S of 11.5 L/m^2^. After the second green phase cultivation, *H. pluvialis* culture was moved in the VBC-PBR to investigate the effect of the two different blue LED lights intensities (low and high: 55 µmol/s/m^2^ equal to 500 lux and 280 µmol/s/m^2^: 2500 lux). The effect of high and low intensity of blue LED light was investigated in terms of chlorophyll content, nutrient consumption and carotenoids, and FAs content, as discussed in the following sub sections. Figure 4a,b show the change in the total chlorophyll content and absorbance in the red spectrum of *H. pluvialis* during the red phase cultivated at high and low intensity of blue LED, respectively. Active *Haematococcus pluvialis* green cells were used for the subsequent red phase under blue light stress conditions for 16 days and chlorophyll content was comparable under high and low light intensity (Figure 4). The increasing absorbance at 750 nm highlights the astaxanthin accumulation. A significantly different absorbance was observed between the 1st and the 2nd switch. After 16 days, the absorbance at 750 nm was 2.5 at high intensity blue LED light (1st switch) while it was equal to 1.5 at low intensity (2nd switch).

Table 2 shows the initial concentration of nutrients during the red phase induction at high light intensity (1st switch) and low light intensity (2nd switch), respectively. The nutrients concentration was higher at the beginning of 1st red phase than second one. In this case, sodium, nitrate, and chloride concentrations were significantly different between the 1st and 2nd red phase.

**Figure 5:**
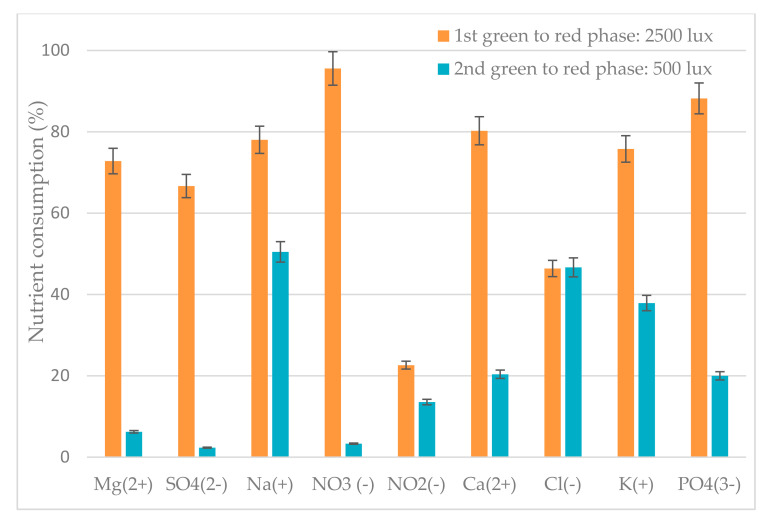
Effect of nutrients and light intensity on nutrient consumption efficiency during the switch from the green phase to the red phase.

Figure 5 represents the nutrients consumption during each switch from green to red phase. Nutrients consumption is evidently high for almost all nutrients during 1st switch at high intensity blue LED. The highest consumption rate was recorded for nitrate, sulfate, and magnesium.

### 3.3. Extraction of Bioactive Compounds from H. pluvialis Red Phase

**Figure 6:**
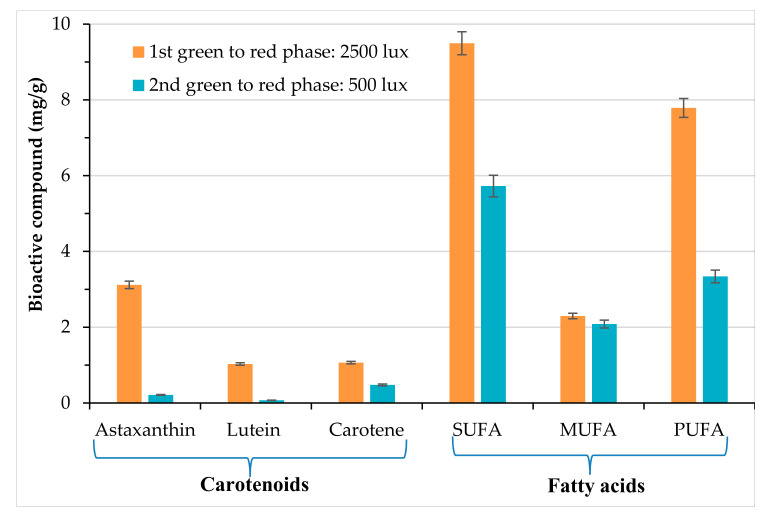
Effect of nutrients and light intensity on production of bioactive compounds in *H. pluvialis* red phase.

The *H. pluvialis* extracts were analyzed by using u-HPLC-DAD for carotenoids and GC-FID for FAs quantification. The data obtained are reported in Figure 6. At high light intensity (2500 lux), the total carotenoids content was ~5 mg/g, of which ~3 mg/g is astaxanthin, while FAs reached ~20 mg/g. At low light intensity (500 lux), carotenoids content was below 1 mg/g and FAs ~8 mg/g. The production of saturated fatty acids (SUFAs), monounsaturated fatty acids (MUFAs), and poly unsaturated fatty acids (PUFAs) under the two light intensities is showed in Figure 6. Saturated and polyunsaturated fatty acids were the highest produced under both high and low light intensity conditions.

The composition of fatty acids is reported in Table 3 and expressed as a percentage of dry weight (% DW). Palmitic acid was the most produced saturated fatty acids under high and low light intensities with a percentage equal to 35.48 ± 1.61 and 44.99 ± 2.19, respectively. Among PUFAs, linoelaidic acid, which is a geometric isomer of linoleic acid, was the most abundant.

**Figure 7:**
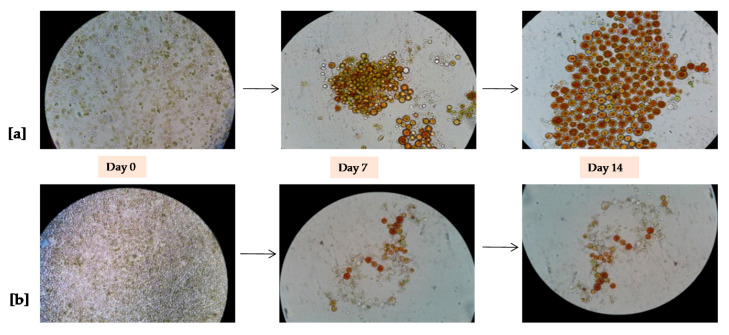
Microscopic observation (Magnification: 400×) of morphological change during the growth of the *H. pluvialis* red phase: (**a**) A first switch from the green phase to the red phase at 2500 lux (280 µmol/photons/s/m^2^) of blue light, and (**b**) A second switch from the green phase to the red phase at 500 lux (55 µmol/photons/s/m^2^) of blue light.

Microscope images were acquired at 0, 7, and 14 days of growth under 2500 and 500 lux light intensities, as reported in Figure 7.

## 4. Discussion 

*H. pluvialis* green cells grew actively during 1st green phase, as demonstrated by chlorophyll-a at 420 nm after 18 days (Figure 2a). During the second green phase, the cells that already underwent a preliminary growth might manifest a low activity and vitality, thus requiring a prolonged time to achieve a biomass concentration comparable to the first cultivation step [30]. However, the chlorophyll content trend was similar to what observed in the first green phase (Figure 2b). During 1st and 2nd green phase, the maximum biomass concentration was 1.7 g/L, which demonstrates that re-using the inoculum has no negative effect. The positive effect of inoculum reuse was also demonstrated by other authors [41,42]. Sun et al., 2017 showed that inoculum replacement enhanced biomass production and astaxanthin accumulation with respect to batch-stage and fed batch-stage. *H. pluvialis* green phase concentration was 1.97 ± 0.08 g/L and 89.17 ± 4.07 mg/L [41]. Witono et al., 2019 observed that the reuse of 37.07% of inoculum and 1.5 mL/L of nutrients promote a high biomass concentration [42]. 

Nutrients concentration and consumption play a crucial role in *H. pluvialis* growth. Although the initial ion concentration was higher in the fresh BG-11 medium for 1st green phase growth, the highest consumption was recorded in 2nd green phase for some nutrients due to their low availability. In 2nd green phase, phosphate and nitrate concentration was ~25% and ~20% lower than their content during the first growth, respectively. This result could be related to a slower rate of biomass accumulation observed after inoculum reuse. Among the nutrients supplied, phosphate was highly consumed during both the first and second growth with a consumption efficiency of 86% and 91%, respectively. This result confirmed the crucial importance of phosphate as an essential element for microalgae cellular constituents such as phospholipids, nucleotides, and nucleic acids, playing a significant role in cellular processes including energy transfer and signal transduction [22]. Furthermore, sodium and nitrate were vastly assimilated during the second green phase with a consumption efficiency of ~94%. Less consumption of magnesium and sulfate were recorded during both green phase stages of with a consumption efficiency below 40%. The results obtained from liquid phase analysis underlined that a longer cultivation time was suitable to enhance the nutrient consumptions efficiency.

The expression of the phytoene synthase gene (psy) was up-regulated in *Haematococcus pluvialis* cells stressed with high light intensity and underwent to the conversion from the green phase to the red phase [57]. The cellular growth started from pear shaped flagellated green vegetative cells to round non-flagellated brown cells and finally transformed into red cysts cells [30]. The *H. pluvialis* red phase was promoted through the use of high intensity blue LED (2500 lux, 280 µmol/photons/s/m^2^) and low intensity blue LED (500 lux, 55 µmol/photons/s/m^2^) in a VBC-PBR with a V/S ratio equal to 11.5 L/m^2^. During the red phase growth (Figure 5), total chlorophyll content gradually declined under stress conditions but absorbance in the red spectrum at 750 nm concomitantly increased up to the 16th day of cultivation. However, after 16 days, total chlorophyll content decreased by 28% and 7% under high and low blue LED light with respect to the initial concentration. This result was due to the adopted stress conditions. The decrease of chlorophyll concentration might directly reflect the status of cellular photosynthetic activities and the response due to stress of high intensity blue light. In addition, high intensity light was suggested for the formation of electro-chemical proton gradient in trans membranes and caused photooxidation of the PS II reaction centers [37]. The stress of blue light showed a positive effect on the absorbance in the red spectrum at 750 nm, which increased by 4.1 and 2.5 times in red phase induced by blue LED light of 2500 lux (280 µmol/photons/s/m^2^) and 500 lux (55 µmol/photons/s/m^2^), respectively. Saha et al. [30] reported that white and green light had a negative impact on red phase induction with a decrease by approximately 26% chlorophyll-a and a negative effect on growth of *H. pluvialis* red phase. Furthermore, our data confirmed that high intensity blue LED light was more effective for the absorbance in the red spectrum (Figure 4a). This result could enhance the production of astaxanthin and others bioactive compounds as fatty acids. Similar observations were reported by Katsuda et al. [39] and Lababpour et al. [40,58]. 

Lower concentration of nutrients was recorded in growth medium for switch b (500 lux) from green to red phase than switch a (2500 lux). Notwithstanding the low concentration, a high content of total chlorophylls resulted in switch b compared to switch a (Figure 4a,b) but this condition was not effective to enhance growth and bio-compound production. Lababpour et al. [58] indeed reported that 10% replacement of medium in fed-batch conditions promoted high concentrations of *H. pluvialis* green cells, but a low concentration of nutrients did not promote significant amounts of astaxanthin. In our work, the growth medium during switch a (2500 lux) from the green phase to the red phase contained a higher amount of nutrients than switch b. The nutrients consumption recorded during each switch from green to red phase, showed that the consumption was more evident during the red phase induction at high light intensity. During this condition, nitrate and phosphate ions were highly consumed, since they are the most important contributors to sustain microalgae growth and for survival strategies [59]. However, low light stress in the switch b from green to red mirrored a 92% lower consumption of NO_3_^−^, while the consumption of Mg^2+^, SO_4_^2^, Ca^2+^, and PO_4_^3−^ was decreased up to 68%. Remarkably, the consumption efficiency of Cl^−^ ions was 46% during the passage from green to red phase at 2500 and 500 lux.

Inoculum reuse during green phase combined with high intensity light blue LED more enhanced the production bio-compounds (carotenoids and fatty acids) in the *H. pluvialis* red phase. Astaxanthin was not the unique carotenoid but lutein and beta-carotene were also produced, although in low amounts. Among FAs, the most abundant were palmitic acid, arachidic acid, cis-11-eicosenoic acid, and linoelaidic acid with a higher concentration obtained at a high intensity blue LED light (2500 lux)., Bio-compounds were extracted from the *H. pluvialis* red phase after mechanical pre-treatment and accelerated solvent extraction through ASE 200. The extraction was performed by using ethanol GRAS solvent at 67 °C and 10 mPa.

The amount of the compounds extracted from *H. pluvialis* red phase stressed at high intensity blue LED light (280 µmol/photons/s/m^2^) was compared with literature data (Table 4). To perform the comparison was difficult since the main experimental conditions, including cultivation system (PBRs, bench scale on flask), working volume, growth medium, light color, and intensity for astaxanthin induction varied in the different studies. The total amount of carotenoids obtained was compared to that observed by Deniz 2020 [60]. Total carotenoid (4.57 mg/g dry weight) was produced in a stirred tank PBR scaled from 2 to 10 L at a constant volumetric power consumption (P/V) [60]. Christian et al. 2018 [61] observed ~3 fold higher astaxanthin yield than this study during cultivation at laboratory scale in 50 mL flask with working volume of 30 mL at 16,200 lux (220 µmol/photons/s/m^2^) and aeration flow rate of ~7 mL/min, and with an energy input 6.5 fold higher than the present work. In this study, 5.21 ± 0.26 and 19.62 ± 0.98 mg/g of total carotenoids and FAs were detected, respectively. The cultivation of *H. pluvialis* supplying high CO_2_ (15%) and exposed to high intensity light could also enhance astaxanthin production [36].

Interestingly, lutein, beta-carotene and FAs extracted in this study during cultivation of *H. pluvialis* at high intensity blue LED light was not observed in the other works [62,63,64]. Recently, Kim et al. 2018 [64] noted that periodic electrical treatment at 25 voltage enhanced the astaxanthin content up to ~21 mg/g, which was 10% increase as compared to those non-treated during the cultivation at 2700 lux. The highest amount of astaxanthin (36.23 ± 5.48 mg/g) was attained during the cultivation with a higher concentration of CO_2_ (15%) and a high light intensity (16,200 lux) as observed by Christian et al. 2018 [61]. Therefore, the integration of different strategies might represent a potential tool to produce astaxanthin and other bioactive compounds. 

## 5. Conclusions

In summary, in this work, a two-stage *H. pluvialis* cultivation was performed with the aim of inducing the production of astaxanthin, lutein and FAs. First, two subsequent growths of *H. pluvialis* green phase were carried out, highlighting that the recycled inoculum led to a slower biomass production with a faster nutrient consumption. The red phase induced from the green one, demonstrated that light intensity plays a crucial role in regulating the stress conditions in *H. pluvialis* for astaxanthin production. Total carotenoids (5.21 ± 0.26 mg/g), including astaxanthin, lutein and beta-carotene, and FAs (19.62 ± 0.98 mg/g), that are precious molecules for health protection and healthy nutrition, were more produced in high intensity light stress condition. In contrast, the production resulted in 2.8-fold lower stress conditions at low intensity light. The comparison with other studies has highlighted the low production yield of the integrated strategy developed. Further studies are surely necessary to improve this weakness through new challenges such as the use of carbon dioxide in the cultivation phase to enhance bio-compound accumulation.

## Figures and Tables

**Table 1:** Nutrient concentrations at the startup of each growth phase for *H. pluvialis* green phase and nutrient consumption during green phase growth.

Nutrients	1st GP-C (mM)	1st GP-Q (mg)	2nd GP-C (mM)	2nd GP-Q (mg)
Mg^2+^	0.22 ± 0.01	36.12 ± 1.63	0.21 ± 0.01	55.12 ± 2.65
SO_4_^2−^	0.12 ± 0.01	92.68 ± 4.63	0.11 ± 0.01	106.71 ± 5.34
Na^+^	10.36 ± 0.26 ^a^	5049.8 ± 126.25	7.98 ± 0.36 ^a^	4842.6 ± 217.92
NO_3_^−^	1.71 ± 0.07 ^b^	2256.8 ± 90.27	1.31 ± 0.06 ^b^	2136.2 ± 96.13
NO_2_^−^	0.10 ± 0.00	56.00 ± 2.52	0.08 ± 0.00	49.64 ± 2.23
Ca^2+^	1.25 ± 0.06 ^c^	597.52 ± 26.89	1.08 ± 0.05 ^c^	513.85 ± 3.12
Cl^−^	2.10 ± 0.08 ^d^	1449.6 ± 57.98	1.66 ± 0.08 ^d^	1359.5 ± 67.98
K^+^	0.36 ± 0.02	223.16 ± 10.04	0.30 ± 0.02	187.9 ± 9.40
PO_4_^3−^	0.17 ± 0.01	386.4 ± 17.39	0.12 ± 0.01	299.06 ± 14.95

GP-C: Green Phase-Concentration, GP-Q: Green Phase-Quantity. Letters ^a^, ^b^, ^c^, ^d^ identicated for each group the statistical significantly value (*p* < 0.05).

**Table 2:** Nutrients concentration at the startup of each switch from green to red phase for *H. pluvialis* red phase and nutrient consumption during red phase growth.

Nutrients	1st RP-C (mM)	1st RP-Q (mg)	2nd RP-C (mM)	2nd RP-Q (mg)
Mg^2+^	0.17 ± 0.01	3.6 ± 0.16	0.13 ± 0.01	0.23 ± 0.01
SO_4_^2−^	0.09 ± 0.00	6.82 ± 0.34	0.07 ± 0.00	0.20 ± 0.01
Na^+^	2.52 ± 0.06 ^a^	54.17 ± 1.35 ^a^	0.46 ± 0.02 ^a^	6.36 ± 0.29 ^a^
NO_3_^−^	0.41 ± 0.02 ^b^	28.99 ± 1.16 ^b^	0.08 ± 0.00 ^b^	0.20 ± 0.01 ^b^
NO_2_^−^	0.05 ± 0.00	0.68 ± 0.03	0.05 ± 0.00	0.35 ± 0.02
Ca^2+^	0.71 ± 0.03	27.55 ± 1.24	0.63 ± 0.03	6.14 ± 0.28
Cl^−^	0.64 ± 0.03 ^c^	12.72 ± 0.51 ^c^	0.29 ± 0.01 ^c^	5.80 ± 0.29 ^c^
K^+^	0.16 ± 0.01	5.71 ± 0.26	0.13 ± 0.01	2.33 ± 0.12
PO_4_^3−^	0.02 ± 0.00	2.24 ± 0.10	0.01 ± 0.00	0.25 ± 0.01

Growth RP-C: Red Phase-Concentration, RP-Q: Red Phase-Quantity. Letters ^a^, ^b^, ^c^ identicated for each group the statistical significantly value (*p* < 0.05).

**Table 3:** Fatty acids (FAs) profile during switch from green to red phase at different intensities of blue LED light.

% FAs	Switch a to 2500 Lux	Switch b to 500 Lux
Butyric acid	0.38 ± 0.02	0.86 ± 0.04
Myristic acid	0.21 ± 0.01	0.02 ± 0.00
Palmitic acid	35.48 ± 1.61	45.43 ± 2.22
Pentadecanoic acid	0.27 ± 0.01	0.62 ± 0.04
Arachidic acid	8.96 ± 0.32	6.28 ± 0.37
Heneicosanoic	0.59 ± 0.03	0.37 ± 0.01
cis-10-Pentadecenoic acid	0.27 ± 0.01	0.37 ± 0.01
Palmitoleic acid	0.38 ± 0.02	1.35 ± 0.07
cis-10-Heptadecenoic acid	0.38 ± 0.02	0.49 ± 0.02
Elaidic acid	2.04 ± 0.11	3.45 ± 0.12
Myristoleic acid	0.21 ± 0.01	0.86 ± 0.04
cis-11-Eicosenoic acid	9.02 ± 0.43	8.62 ± 0.37
Linolenic acid	0.05 ± 0.00	0.00 ± 0.00
Linoelaidic acid	40.79 ± 1.83	31.27 ± 1.60
γ-Linolenic acid	0.97 ± 0.05	0.00 ± 0.00

**Table 4:** Comparison of literature data with our study for production of bioactive compounds from *H. pluvialis*.

Cultivation Conditions				Production of Bioactive Compounds (mg/g)				Reference
LI (Lux)	CT (Days)	WV (mL)	AFR (mL/min)	Astaxanthin	Lutein	β-Carotene	Fatty Acids	
7290	15	400	120	~4	na *	na	Na	[35]
5832	15	400	120	~6.4	na	na	Na	[36]
5400	4	30	∼7	8.87 ± 2.7	na	na	Na	[61]
16,200	4	30	∼7	9.27 ± 1.0	na	na	Na	[61]
2500	14	1200	50	3.12 ± 0.1	1.03 ± 0.1	1.07 ± 0.1	19.62 ± 0.6	This study

LI: Light intensity; CT: Cultivation time; WV: Working volume; AFR: Aeration flow rate; na *: data was not available.
